# Formulation of a protocol to evaluate the aerobic granulation potential (AGP) of an inoculum

**DOI:** 10.1016/j.mex.2022.101710

**Published:** 2022-04-22

**Authors:** Dayana Grisales Penagos, Jenny Rodríguez Victoria, Mateo Villarraga Manrique

**Affiliations:** ECCA Group, School of Engineering, Universidad del Valle, Santiago de Cali, Colombia.

**Keywords:** Aerobic granules, Inoculum, Protocol, AGP, Aerobic Granulation Potential, EPS, Extracellular Polymeric Substance, SBR, Sequential Batch Reactor, SVI, Sludge Volumetric Index, VER, Volumetric exchange ratio, DO, Dissolved Oxygen, H/D, Height Diameter Ratio, F/M, Food Microorganism Relationship, COD, Chemical Oxygen Demand, PVC, Polyvinyl Chloride, OLR, organic loading rate, HRT, Hydraulic retention time

## Abstract

This paper proposes and develops a protocol for measuring the aerobic granulation potential of sludge, aiming to provide an affordable and simple alternative that can facilitate the development of aerobic granulation technology. In this sense, the protocol comprises a set of parameters and considerations that interact to create a controlled environment and stimulate cell population clustering. All of this is done in the context of procedural simplicity, low cost, and the speed at which results are obtained. The protocol is essentially a three-stage method: preparation of the substrate, adaptation of the inoculum, and implementation of the protocol. Simple parameters were measured to evaluate the granulation process: SVI, settling velocity, and morphological parameters. The protocol was validated according to optimal ranges and criteria previously established in the literature. For this purpose, an activated sludge inoculum from a domestic wastewater treatment plant was submitted to the protocol, obtaining an optimal response of the biomass (SVI_5_ =13.90 mL g^−1^, settling velocity= 25,79 m h^−1^, Diameter > 0.2 mm) in a relatively short time (7 d). The results show that this protocol can constitute a tool for evaluation and decision-making using traditional laboratory equipment and is applicable at different scales.

## Graphical abstract

**Figure:**
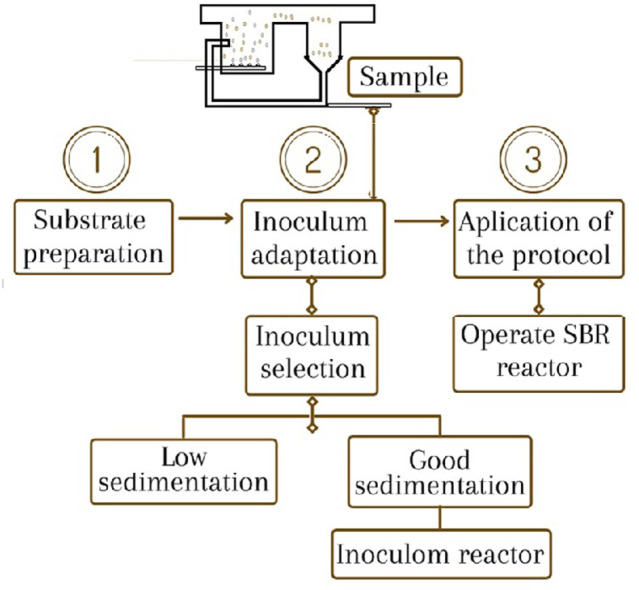

Specifications table

**Table:** 

Subject Area:	Environmental Science
More specific subject area:	Biological wastewater treatment
Protocol name:	Aerobic granulation potential (AGP)
Reagents/tools:	*Reagents* Determination of chemical oxygen demand (COD): Silver sulfate (Ag_2_ SO_4_). Sulfuric acid (H_2_SO_4_, 96-97%). Mercury sulfate (HgSO_4_). Potassium dichromate (K_2_ Cr2O_7_). Potassium hydrogen phthalate (KPH) Substrate: Sodium acetate (Na_3_CH_3_COO) Micro- and Macronutrients: Dipotassium phosphate (K_2_HPO_4_) Monopotassium phosphate (KH_2_PO_4_) Sodium chloride (NaCl) Magnesium chloride hexahydrate (MgCl_2_.6H_2_O) Dehydrated calcium chloride (CaCl_2_.2H_2_O) Ferric chloride hexahydrate (FeCl_3_.6H_2_O) Magnesium chloride tetrahydrate (MnCl_2_.4H_2_O) Cobalt chloride hexahydrate (CoCl.6H_2_O) Calcium chloride dihydrate (CaCl_2_.2H_2_O) Anhydrous zinc (ZnCl_2_) Copper chloride dihydrate (CuCl.2H_2_O) Trioxoboric acid (H_3_BO_3_) Sodium selenite pentahydrate (Na_2_SeO_3_.5H_2_O) Nickel chloride hexahydrate (NiCl_2_.6H_2_O) *Equipment and materials* Volumetric balloons (10 mL, 500 mL, and 1000 mL) Beaker (500 mL and 1000 mL) Test tube (1000 mL) Imhoff cones 0.45 µm glass microfibre filter membrane COD tubes Micropipettes (200 µl and 1000 µl) Centrifuge pH meter Dispensers (5 mL) Vacuum pump Analytical balance. COD digester Fume hood Oven (103°C and 120°C) Spectrophotometer *Equipment and specific materials of the protocol* Column type reactor, preferably transparent (glass or PVC, 280 mL) Microscope Flowmeter Aerator Camera Arduino breadboard, accessories, and Arduino 4-channel relay module Neubauer chamber Peristaltic pumps
Experimental design:	The proposed protocol establishes a set of operational parameters, ranges, and relevant considerations that stimulate aerobic granulation in the inoculum to evaluate and determine its transformation capacity to granules in a controlled environment.
Trial registration:	*NA*
Ethics:	*NA*
Value of the Protocol:	The protocol is a novel tool for obtaining early results and decision-making about inoculum sources for aerobic granulation technology. The protocol can be simply and affordably implemented at different scales, with few economic, physical, and temporal resources. The protocol has a flexible configuration, and once the granulation potential has been established, the operational conditions can be adjusted to improve results.

## Background

Aerobic granulation is a novel and innovative technology that has gained increasing interest due to its potential in treating wastewater. Since the first laboratory-scale sequencing (SBR) batch reactors were used in the mid-1990s [37], several studies have found that aerobic granulation has advantages such as a denser and stronger aggregate structure, better settling characteristics, higher microorganism concentration, and efficient contaminant removal. In effect, aerobic granulation has begun to be regarded as a profitable technology for treating large volumes of wastewater in smaller reactors, and this process applies to a wide range of types of wastewater [19], [59]. Moreover, some researchers have suggested that the application of aerobic granulation instead of conventional activated sludge systems could reduce energy consumption by about 68% [47].

Aerobic granules are defined as groups of self-immobilized microorganisms that occur in dense and compact spheres [51]. Each sphere of biomass can be composed of several biological layers as a function of oxygen penetration and microintegrating populations of multifunctional microorganisms [22], [34], [42], [68]. Cell-to-cell adhesion occurs in biological, physical, and chemical phenomena [34].

When research related to granulation is carried out, an inoculum that is not adapted to the conditions of its new environment can lead to long start-up periods before mature granules can be obtained. Therefore, this represents economic, physical, and time costs, mainly if the capacity of the inoculum to develop into aerobic granules is unknown. Since its successful culture was first reported, many studies have described different methodologies for obtaining aerobic granulation [50], [64]. However, these methodologies require significant resources and are not sufficiently clear or easy to implement, and more knowledge regarding the underlying mechanisms behind granulation is needed. Furthermore, the main disadvantage of aerobic granulation is the long start-up period, so it should be eliminated. Measures should be taken for rapid granule formation by selecting appropriate strains that favor microbial aggregation.

Based on this scenario and the need to find affordable alternatives to facilitate and promote the technological development of aerobic granulation, the Aerobic Granulation Potential (AGP) protocol was proposed. The AGP protocol is a set of parameters and methods for creating a controlled environment to stimulate biomass aggregation. By performing simple and fast-running tests such as settleability and morphological parameters, it is possible to quickly assess the viability of clustering the cell population in the inoculum under study.

The PGA protocol is an evaluation and decision-making tool applicable at different scales. Its implementation is simple and low-cost, and it evaluates in a shorter time the possibility and capacity of the formation of aerobic granules from the different inoculum.

Through a comprehensive analysis of available literature [6], [7], [17], [22], [28], [55], [58], [63], [66], [68], optimal ranges of operational variables for the AGP protocol were selected, which were approved by several tests.

Initially, factors such as the substrate, the configuration of the reactor, and the methods to ensure the proper reactor performance are determined. Once these factors are verified, the steps constituting the AGP protocol are established. After establishing the AGP protocol, it is validated by comparing the size and structure of the obtained granules with the results of previous investigations.

**Fig. 1:**
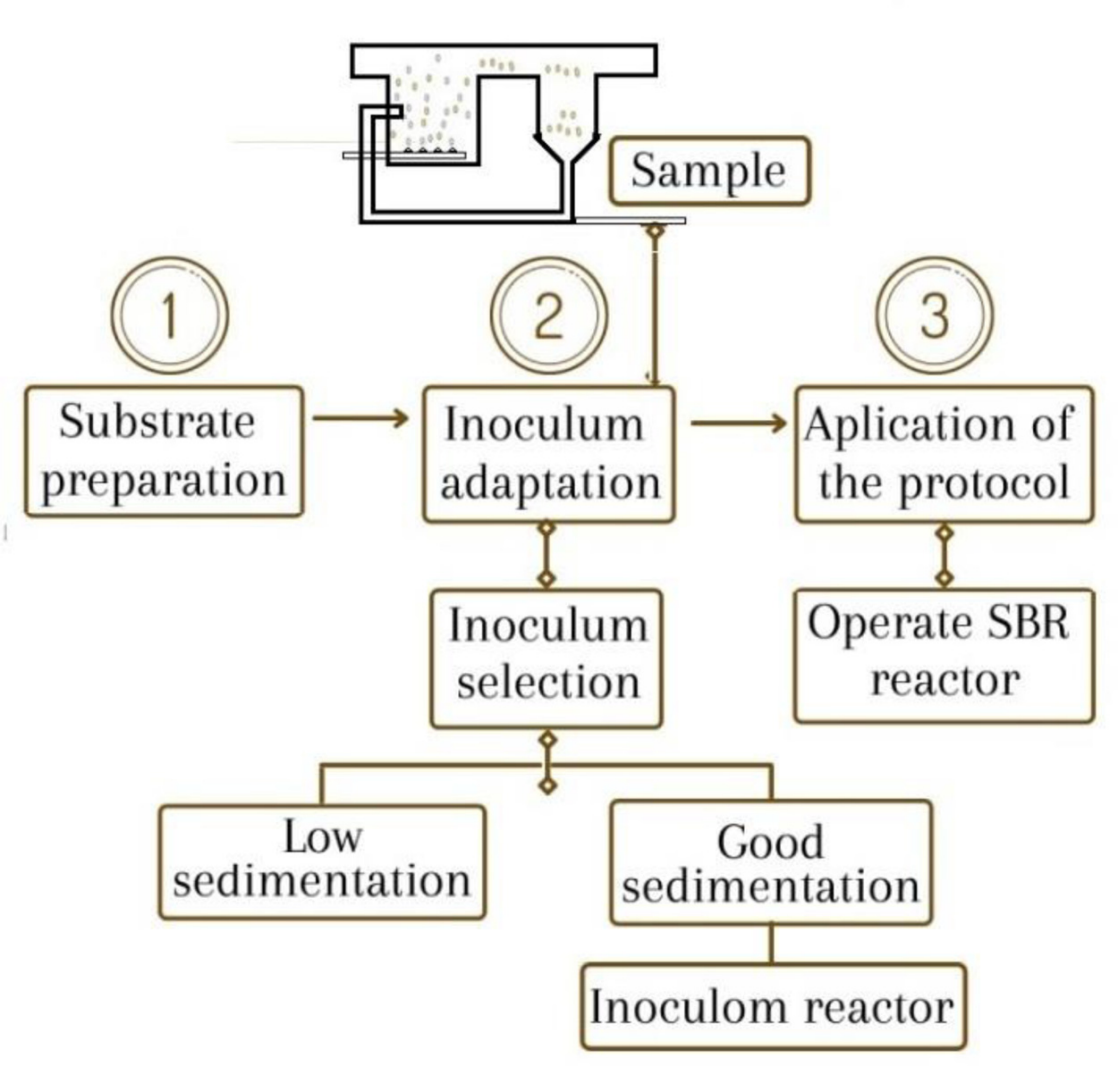
AGP protocol application stages.

**Fig. 2:**
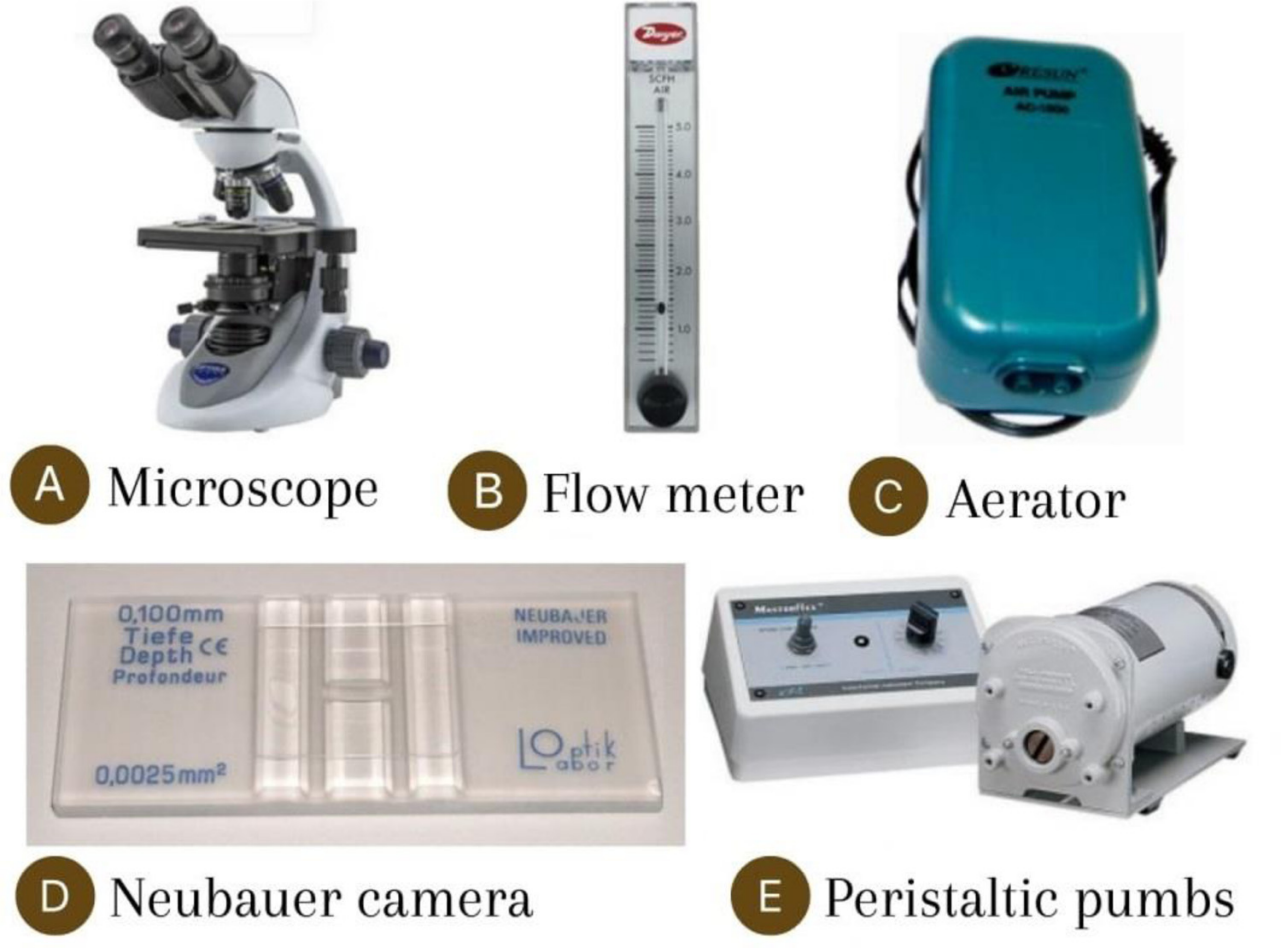
AGP protocol equipment.

The AGP protocol is essentially a three-stage method: preparation of the substrate, adaptation of the inoculum, and implementation of the protocol as shown in Fig. 1. Fig. 2 shows the equipment used in the AGP protocol.

## Substrate preparation

In contrast to other widely used substrates such as glucose, different studies have emphasized acetate as an efficient carbon source for growing granules. Acetate promotes the production of extracellular polymeric substances (EPS) [61], and granule-forming microorganisms (*Methanosarcina* and *Methanothrix*) prefer it [39]. The granules derived from acetate appeared to be more compact and had better sedimentation characteristics [63]. Furthermore, acetate is considered a biodegradable substrate that is rapidly assimilated by granule-forming microorganisms [46], thus making it an option to determine the effect of the inoculum without the intervention of the substrate composition.

Consequently, sodium acetate (Na_3_CH_3_COO) was selected as the substrate, and it was enriched with mineral salts and micronutrients, whose compositions are presented in Table 1 and Table 2. The concentration of the substrate solution will depend on the organic loading rate (OLR) applied in the test.

**Table 1:** The concentration of mineral salts.

Composite	Formula	Concentration (gL^−1^)	Volume (mL.L^−1^AR)
Dipotassium phosphate	K_2_HPO_4_	6	3.75
Dipotassium phosphate	K_2_HPO_4_	6	1.04
Sodium chloride	NaCl	12	20.80
Magnesium chloride hexahydrate	MgCl_2_.6H_2_O	2	3.50

Source: Vazoller [67].

**Table 2:** Concentration of micronutrients.

Composite	Formula	Concentration (g L^−1^)
Ferric chloride hexahydrate	FeCl_3_.6H_2_O	1.35
Magnesium chloride tetrahydrate	MnCl_2_.4H_2_O	0.10
Cobalt chloride hexahydrate	CoCl.6H_2_O	0.02
Calcium chloride dihydrate	CaCl_2_.2H_2_O	0.10
Anhydrous zinc	ZnCl_2_	0.10
Copper chloride dihydrate	CuCl.2H_2_O	0.02
Trioxoboric acid	H_3_BO_3_	0.01
Sodium chloride	NaCl	1.00
Sodium selenite pentahydrate	Na_2_SeO_3_.5H_2_O	0.03
Nickel chloride hexahydrate	NiCl_2_.6H_2_O	0.12

Source: Touzel and Albagnac [65].

## Preparation of the inoculum

**Fig. 3:**
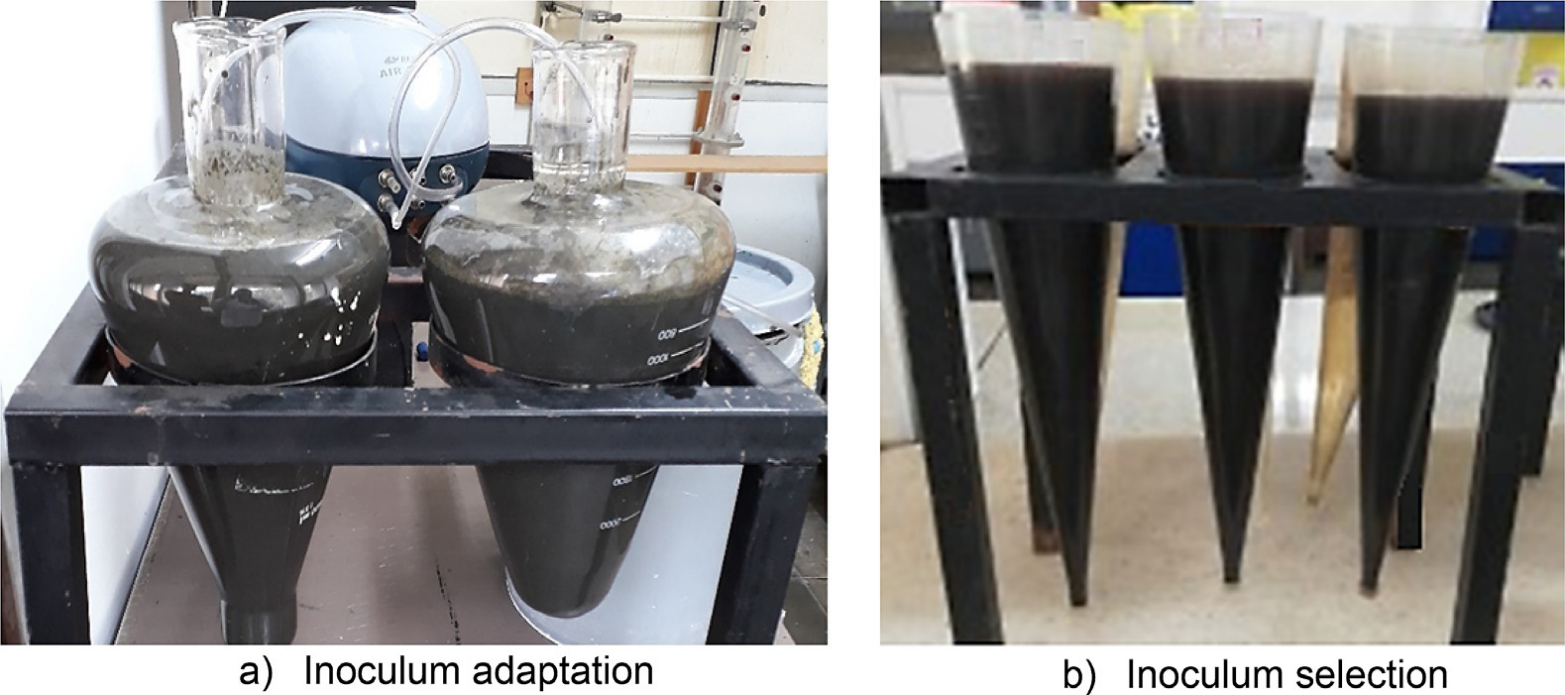
Preparation of the inoculum.

Before the reactor inoculation begins, the inoculum must be exposed to an adaptation process so that the microorganisms assimilate the new substrate and maintain maximum microbial diversity. The adaptation process involves putting the inoculum in contact with the substrate at a 50/50 ratio in volume, using an aerated conical vessel (Fig. 3a). The assimilation of the substrate is determined by monitoring the reduction of the chemical oxygen demand (COD) until it reaches a value greater than 40%.

After adaptation, the inoculum is subjected to a selection process proposed by Sheng et al. [54] with some modifications. The modified selection process consists of filling a 1 L Imhoff cone with the adapted inoculum, stirring gently, and allowing the inoculum to settle for two minutes. Then, the supernatant is removed, and the remaining inoculum is the one that will be used to inoculate the reactor (Fig. 3b). This process is repeated until the required inoculation volume is achieved. The total volume of the inoculum is homogenized to determine the sludge concentration with a TSS and VSS test according to standard methods [14].

Activated sludge has been the most widely used inoculum in aerobic granulation [2], [3], [23], [56], [69], [70], despite its bacterial communities being hydrophilic [75]. This fact is because of its extraction ease, high density, and microbiological diversity. Accordingly, the inoculum used was activated sludge from a municipal wastewater treatment plant.

## Application of the protocol

The application of the protocol begins with the start-up of a sequential batch reactor (SBR) used as the experimental unit. This reactor type has been widely used and is highly recommended for aerobic granulation because a discontinuous operation is advantageous to the process. Furthermore, the SBR creates an adverse environment and stress conditions for the microbiota that lead to aggregation as a survival mechanism [63], promoting the selection of granular biomass and the wash-out of flocculent or filamentous microorganisms [26].

**Fig. 4:**
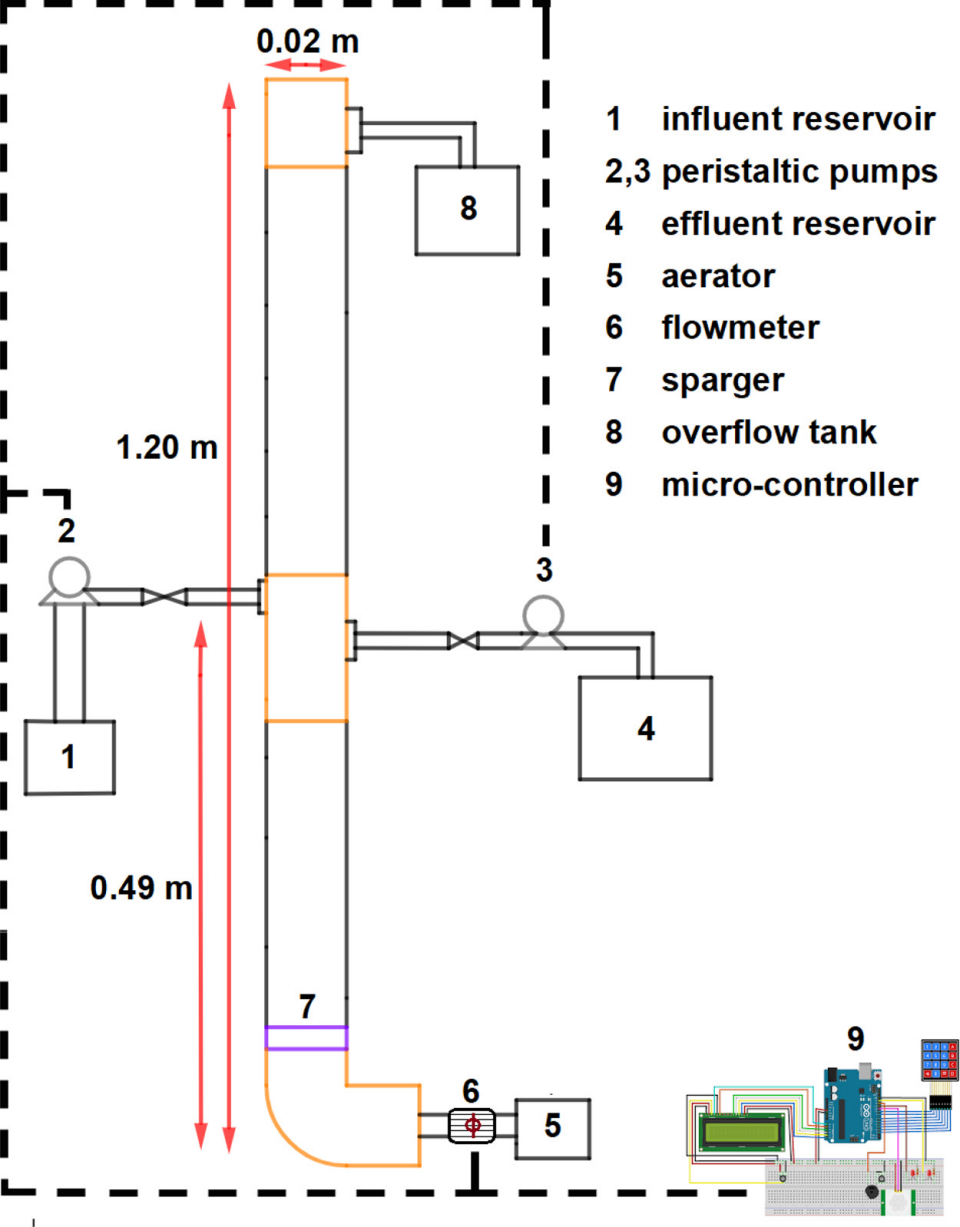
General diagram of the SBR column reactor.

Experimental unit. The fibber glass reactor has a working volume of 280 L with an internal diameter of 0.02 m and an effective height of 0.98 m (Fig. 4). Air is introduced through a diffuser by an air pump at the bottom of the reactor. The reactor is equipped with influent and effluent ports located at midheight of the reactor, yielding a volumetric exchange rate of 50%. The upflow shear force is adjusted by changing the airflow rate.

Operational parameters. Table 3 shows the parameters and values used for the SBR operation. Because the research on aerobic granulation is relatively recent, many aspects are not fully understood and require further investigation. Therefore, in some parameters, a consensus was made on the more recurring information from the available literature that showed good or excellent results in the formation of aerobic granules.

**Table 3:** Parameters used for the AGP protocol.

Parameter	Value	Units	Reference
Reactor Type	SBR	-	[41], [53]
H/D ratio	≥6:1	-	Yilmaz et al. [72], Liu y Tay [35], Tao et al. [62], Bassin et al. [6]
Cycle time	3	H	Abdullah et al. [1], Kong et al. [23], Devlin et al. [13]
Number of cycles per day	8	-	
HRT	6	H	
Sedimentation time	15–5–2	min	McSwain et al. (2004),Sheng et al. [54], Corsino et al. (2017)
Volumetric exchange ratio - VER	50	%	Chen et al. [9], Liang et al. [30], Devlin et al. [13], Bassin et al. [6]
Discharge time	1	min	Kang y Yuan [21]
F/M	0.64	gDQO (gSSV)^−1^	Li et al. [29], Wu et al. [71]
Substrate	Acetate	-	Sun et al. [61], Kong et al. [23], Liu y Tay [35]
COD of the substrate	1500	mg.L^−1^	
Organic loading rate -OLR	6.00	kg.m^−3^.d^−1^	Moy et al. [40], Khan et al. (2011), Liu y Tay [35]
Aeration time	163–173–176	min	Cui et al. [11], Liang et al. [30]
Aeration rate	2.40 to 3.00	cm s^−1^	Chen et al. [10], Lee et al. [26],Kang y Yuan [21]
DO	0.50 to 6.00	mg L^−1^	Morais et al. [38], Bassin et al. [6]
pH	6.50 to 8.00		[46], Wu et al. [71]
Temperature	20 to 25	°C	Niu et al. [42], Bassin et al. [6]

The SBR is operated with sequential cycles until it reaches granulation. Each cycle consists of four sequential stages: filling (feeding), aeration (reaction), settling (solid-liquid separation), and discharge (effluent withdrawal). The cycle time is 3 hours, and the cyclic operation is achieved by a micro-controller (Arduino Uno R3). Due to the need to adjust the settling time as the granulation process advanced, the protocol development was divided into three phases, each with a specific duration. The configuration of the cycles and phase times are shown in Table 4.

**Table 4:** Times of the operation cycle.

Stage	Time (min)				Duration (d)
	Feeding	Aeration	Settling	Discharge	
Phase 1	1	163	15	1	8
Phase 2	1	173	5	1	1
Phase 3	1	176	2	1	2

Analytical procedure. In the AGP protocol, three issues are monitored to evaluate the development of granulation and treatment capacity: i) physicochemical properties of influent and effluent, measurement of pH, dissolved oxygen, temperature, and COD; ii) sludge settleability, assessed by volumetric sludge index and settling velocity, and iii) sludge morphology, analyzed by roundness, circularity, and equivalent diameter. The analyses are performed based on the methodologies shown in Table 5.

**Table 5:** Methodologies implemented in the AGP protocol.

Sample	Variable	Methodology
Influent, effluent, mixed liquor	pH Temperature Dissolved oxygen COD	Standard Methods (APHA et al., 2005)
Substrate	COD	
Inoculum Biomass Granules	Sludge volumetric index (SVI)	Standard Methods (APHA et al., 2005)
	Settling velocity	Su and Yu. [60] and Song et al. [57]
Biomass Granules	Equivalent diameter Circularity Roundness	Beun et al. [7]

The SVI results were contrasted with the SVI scale proposed by [16], as shown in Table 6.

**Table 6:** Relationships between the SVI and the compaction and settling characteristics.

SVI range (mL.g^−1^)	Compaction and settling characteristics
<80	Excellent
80-150	Moderate
>150	Poor

The settling velocity was determined using the methodology proposed by Su and Yu [60] and Song *et al*. [57] consisting of the following steps:

1. Use aerobic granules with spherical characteristics.
2. Fill a 1 L graduated cylinder with tap water.
3. Pour gently and in a steady flow the previously selected granules on the surface of the water.
4. Allow the granules to settle.
5. Start the timer immediately after pouring the granules. Record the time from the beginning to the end of the route.
6. Calculate the settling velocity from Eq. 1.
  (1) $$V_{s}=\frac{L}{t}$$

where *V_s_* = sedimentation velocity of a granule (ms^−1^), *L* = distance traveled by the granule (m), and *t* = time taken by the granule to complete the journey to the bottom (s).

A positive result is considered when the settling velocity is ≥ 10 ms^−1^ [5], [41], [66].

Biomass monitoring. Because the granulation process involves a gradual change in the configuration of microbial aggregations from a flocculent structure to a denser and more compact granule [34], [54], [57], [63], it is necessary to identify the morphological characteristics and biodiversity to establish the capacity and properties present in the aggregations inside the reactor. Determining the morphology of sludge involved the following steps:

1. During the aeration phase, sample 50 mL from the reactor bottom.
2. Make photographic records of the sample under a microscope equipped with a Neubauer camera. Photographs must be taken with the same approach and device.
3. Process the photographic record in graphic design software. Use an image captured from the Neubauer camera as a contrast plane, which functions as a measurement pattern on the photographs at different objectives, 4X, 10X, 40X, and 100X (Fig. 5).

**Fig. 5:**
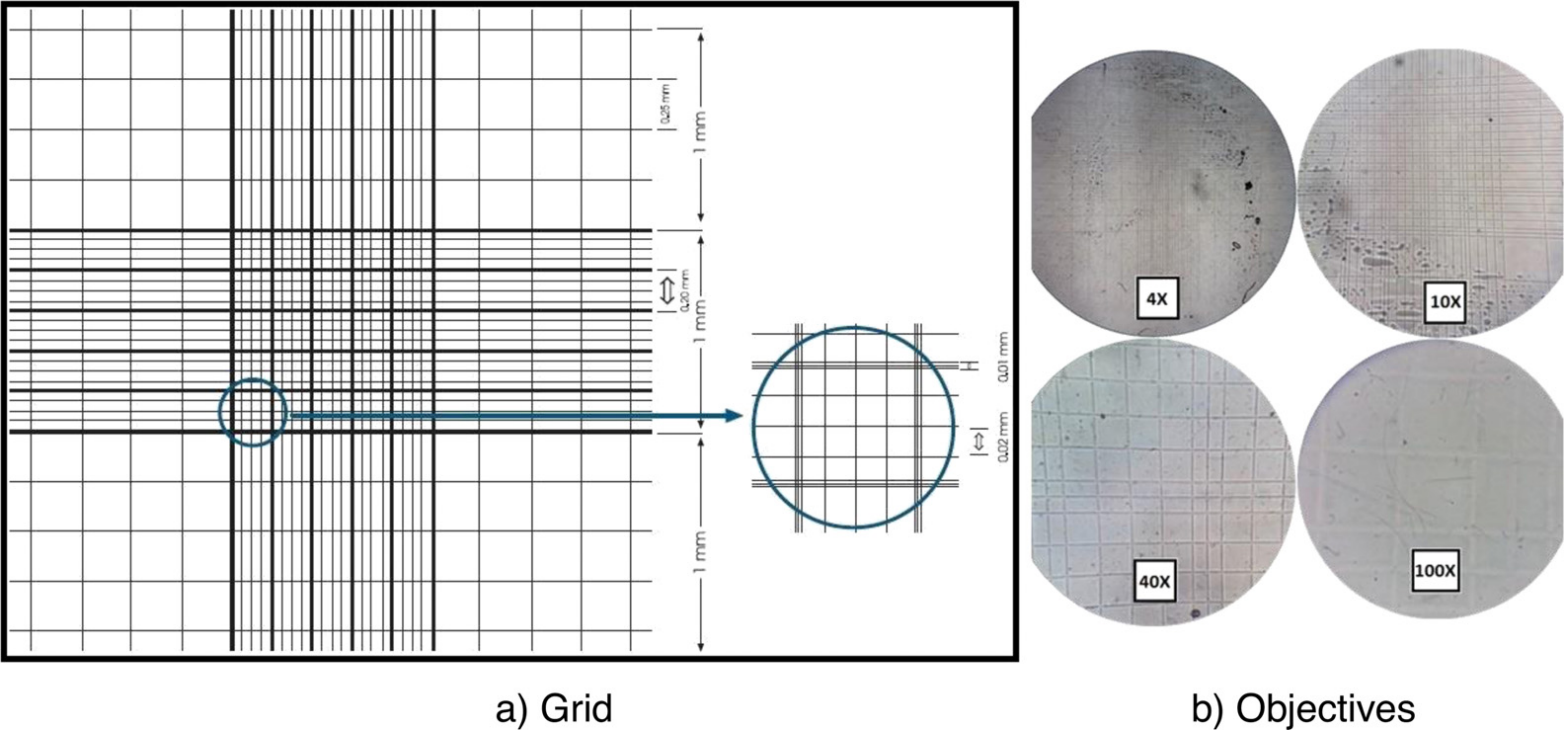
Grid photographs of different objectives of the Neubauer chamber.
4. Measure equivalent diameter, circularity, and roundness.

The equivalent diameter corresponds to the equivalent diameter (Deq) value that the cell aggregation would occupy when projected on a plane, which is determined by Eq. 2 [20].

(2) $$Deq=2\;\times \sqrt{\frac{Area}{\pi }}$$

where Deq= the equivalent diameter of the two-dimensional projection (*µ* m) and Area = the extension of the aggregation surface, obtained using graphic design software (*µ* m ^2^).

Aerobic granules with a diameter greater than 0.20 mm are used as a positive indicator ([41], [66], and [49]).

Through roundness and circularity, it is possible to characterize the degree of maturity and development of cellular aggregations, both in flocs and in aerobic granules. Circularity refers to the degree of geometric regularity of the surface of the particle in a two-dimensional plane and is calculated by Eq. (3) [24]. Roundness is the degree of circularity established by the maximum and minimum measurements from the center of the particle as determined by Eq. (4) [24].

(3) $$Circularity=\;\frac{4\;\times \pi \times A}{{\left(L_{O}\right)}^{2}}$$

Circularity=1→Circle;Circularity=0→Line

(4) $$R_{O}=\;\frac{r_{min}}{r_{max}}$$

$$R_{O}=1\to C\prime ırcle;R_{O}=0\to Line$$

where A= the projection surface of the particle on a plane (µm *2),* $L_{O}$ = the perimeter of the aggregate or equivalent to the length of the edge of the particle (µm), $R_{O}$ = the roundness of the particle (adim), $r_{min}$ = the minimum measured particle radius (*µ* m) and$\;r_{max}$ = the maximum measured particle radius (*µ* m).

The acceptance value for granular formations in terms of circularity and roundness is 0.70 [7], [15], [20], [28], [44], [60].

## AGP protocol procedure

1. Prepare the macro-and micronutrient stock solutions according to the established concentrations and doses. Keep refrigerated.
2. Determine the concentration of COD in the sodium acetate solution to be used (substrate).
3. Verify SBR System Operation and Equipment.
4. Perform inoculum adaptation. Homogenize the adapted inoculum and take a sample. Perform a microscopy examination and determine the concentration of solids. From a centrifuged sample, take the supernatant to measure the COD. Make at least three measurements during the process: initial, intermediate, and at 24 hours.
5. Inoculate the reactor with the required volume to ensure the established F/M ratio.

Note: In the first cycle of operation, add 50 mL of inoculum to the seeding volume to measure the concentration of solids.

1. Monitor COD (influent, effluent, and mixed liquor), pH, dissolved oxygen (DO), and morphology (biomass) when a change in the biomass is noticed.

Note: Sampling must be performed during the aeration stage because the complete mixture is maintained inside the reactor.

2. Gradually reduce the settling time as follows: 15 minutes for the first eight days, 5 minutes for the next day, and 2 minutes for the last two days.

Note: The proposed settling times can be adjusted according to the settleability characteristics of the granules being formed.

1. Continue reactor operation for approximately 11 days or less if favorable sludge aggregation is observed.
2. Empty the entire contents of the reactor into a container at the end of the operation, homogenize, and take a sample to determine the sludge volumetric index (SVI), settling velocity, and morphology (Fig. 6).

**Fig. 6:**
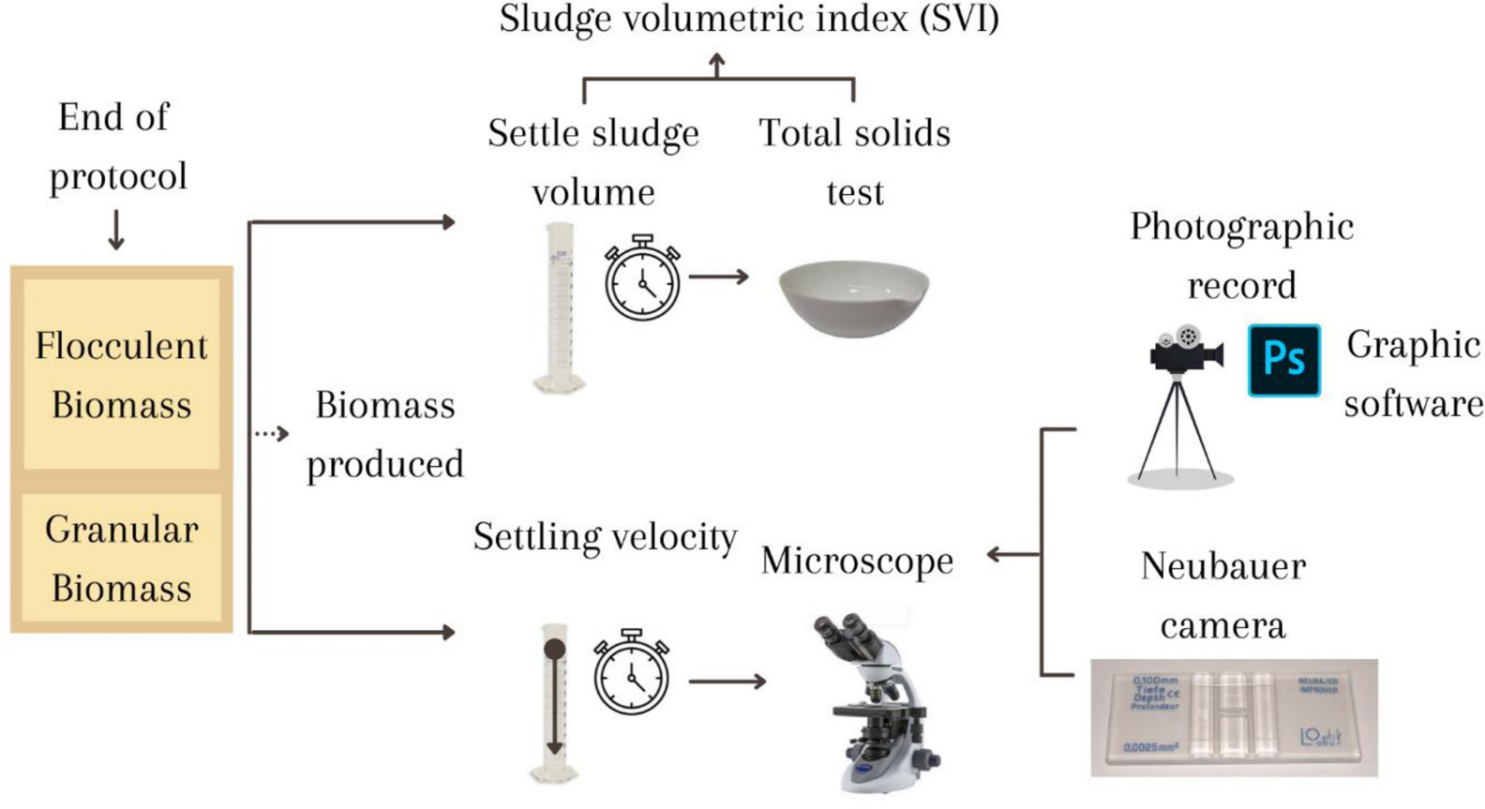
Diagram of the process for determining settling characteristics and morphology.
3. Record, process, and assess the data generated, including digital photography. To reduce the margin of error for photo observations, use the same photographic camera and the same picture characteristics.
4. Compare the results with the criteria previously established in the available literature to determine whether aerobic granules were obtained.

### Validation of APG protocol

A validation process was performed to confirm the applicability of the AGP protocol. For this purpose, an activated sludge inoculum from a domestic wastewater treatment plant in Cali-Colombia was submitted to the AGP protocol. The results of the monitored parameters are presented below.

Physicochemical characteristics and OLR. During the application of the protocol, the pH in the mixed liquor was maintained within the established range (6.50 to 8.50). The behavior of DO showed variations and had minimums of up to 0.20 mg L^−1^, but it did not affect biomass transformation. Although the OLR values were lower than those established in the protocol, the biomass response was optimal. Table 7 shows the values found.

**Table 7:** Physicochemical characteristics and OLR.

Variable	Value
pH	7.34 to 7.99
Temperature (°C)	24.80 ± 0.21
OD (mg L^−1^)	2.00 ± 2.52
OLR (kg (m^3^.d)^−1^)	5.40 ± 0.19

**Fig. 7:**
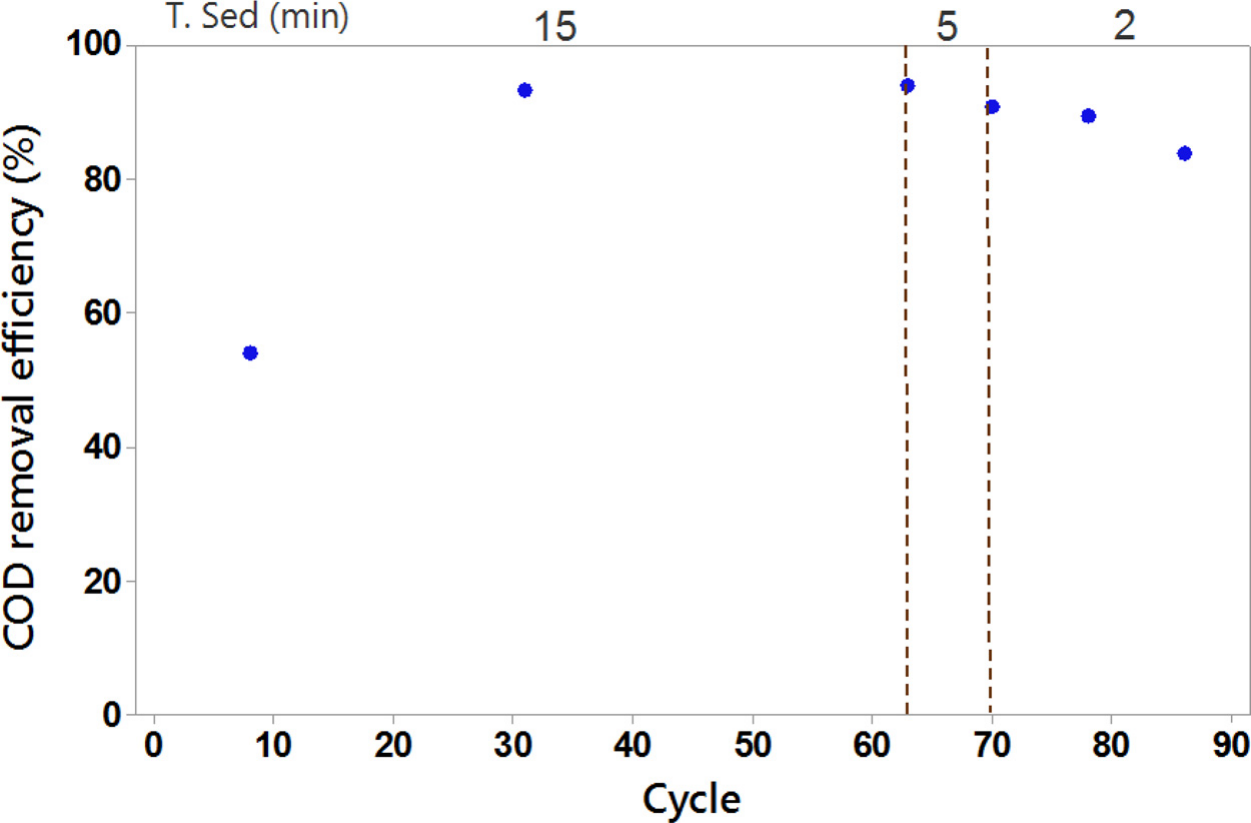
COD reduction during reactor operation.

The reactor performance was improving continuously in terms of COD removal efficiency during the reactor operation (Fig. 7). This improvement became obvious after around 10 cycles of operation. After cycle 25, the COD removal efficiency reached over 85%, demonstrating the speed and effectiveness of the system in reducing COD. Furthermore, the decrease in settling time affected COD removal because of the wash-out of biomass with low sedimentation characteristics. Despite that, the settling time reduction resulted in a strategy to incentivize the selection of biomass.

Sedimentation characteristics. The SVI_5_, SVI_30_, and settling velocity were performed to characterize the biomass settleability. Table 8 shows biomass with excellent settling properties. When the SVI30 obtained at the beginning and final reactor operation are compared, a significant transformation is observed, changing from flocculent to more settleable biomass. Additionally, the similarity of the values of IVL5 and IVL30 is characteristic of aerobic granules [66], corroborating the degree of granulation achieved.

**Table 8:** Biomass settleability at final of APG test.

Variable	Value
SVI_5_ (mL.g^−1^))	13,89
SVI_3_ (mL.g^−1^)	13,33
Settling velocity (m.h^−1^)	25,79

The settling velocity obtained demonstrates the adequate development of the granulation process when compared to that indicated by Liu *et al.* [33], who stated that the settling velocity of a granule should not be less than 8 m h^−1^. Other studies report values between 10 and 15 m h^−1^ [8], [43].

**Fig. 8:**
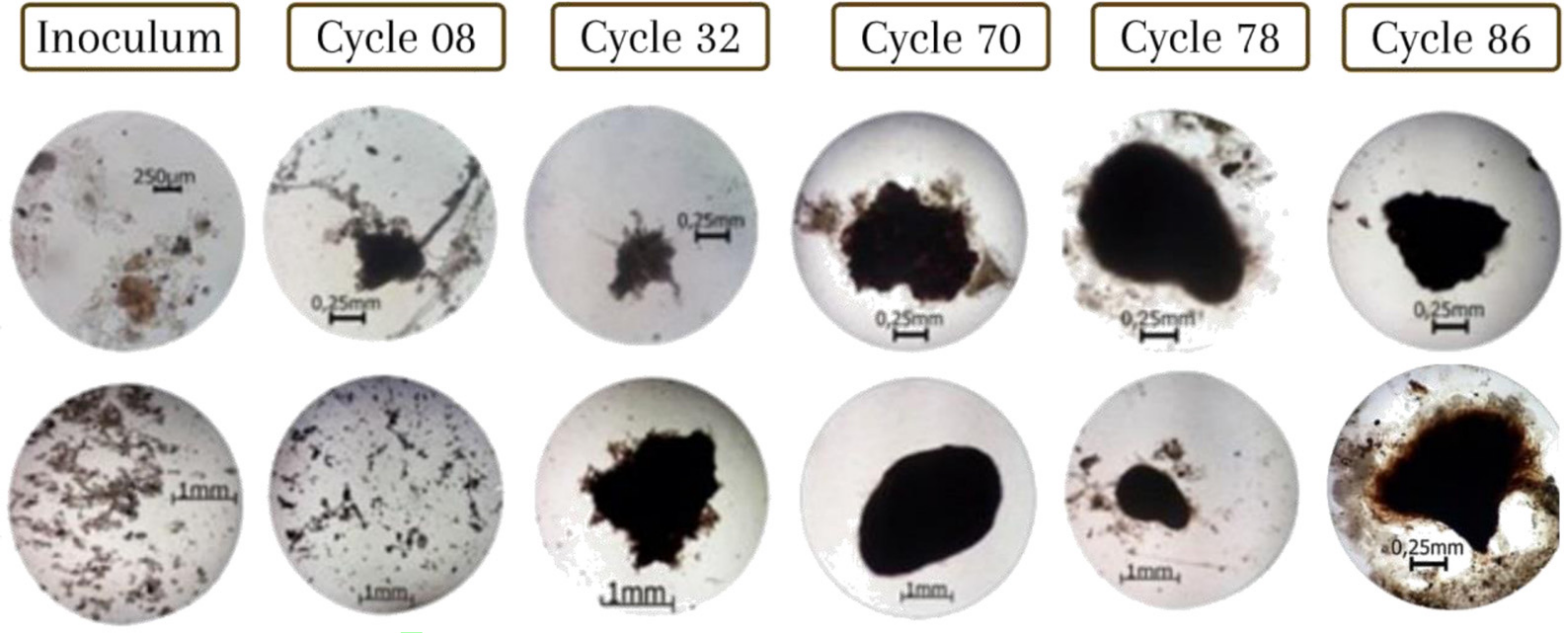
Development of aggregates during experimentation.

Morphological characteristics. The morphological change of the flocs to aggregates was visible in the APG test ( Fig. 8). The observed evolutionary process was consistent with that reported by different researchers, such as Hailei *et al.* [18]: fragmentation of cellular aggregates, collision, union, polish, compaction, and maturation of the aerobic granule.

The inoculum consisted mostly of flocs with sizes between 100 and 150 µm (measured as equivalent diameter), corresponding to flocs of activated sludge [25], [73]. After 8 cycles, the flocs decreased in size to values with a median of 60 µm due to the shear force generated by aeration, which impacted the weak structures that make up the flocs [31], [45], [63].

In cycle 32, the sizes of aggregations (median 50.37 µm) were similar to those of cycle 8, but two types of aggregations were identified: flocs and dense aggregates. The flocs were irregular structures formed of filamentous microorganisms. According to available literature [7], [19], [27], [36], [74], filamentous microorganisms function as structural skeletons where cells aggregate and eventually develop into aerobic granules. The dense aggregates were spherical, surrounded by biofilm, and classified as an intermediate between flocs and aerobic granules.

In cycle 70, there was a significant increase in the equivalent diameter (median 1362.80 µm) corresponding to the conformation of aggregates with greater weight and definition. The aggregations found showed different colorations: the densest were dark or brown, and the flocculent was light or whitish. The morphologies observed are similar in shape and color to those obtained by different investigations [4], [6], [12], [35], [60].

In cycles 78 to 86, the sedimentation process was evident inside the reactor due to the aggregates with greater weights and sizes that were formed. Spherical geometries and sizes up to 3 mm are highlighted. Granules were identified, which could be defined as intermediate agglomerates with an indefinite geometry and with a proliferation of filamentous microorganisms on the surface. The available literature reports that the proliferation of filamentous microorganisms is a side effect of shear force reduction, so aggregations become spongy [9], [13], [31], [63].

**Fig. 9:**
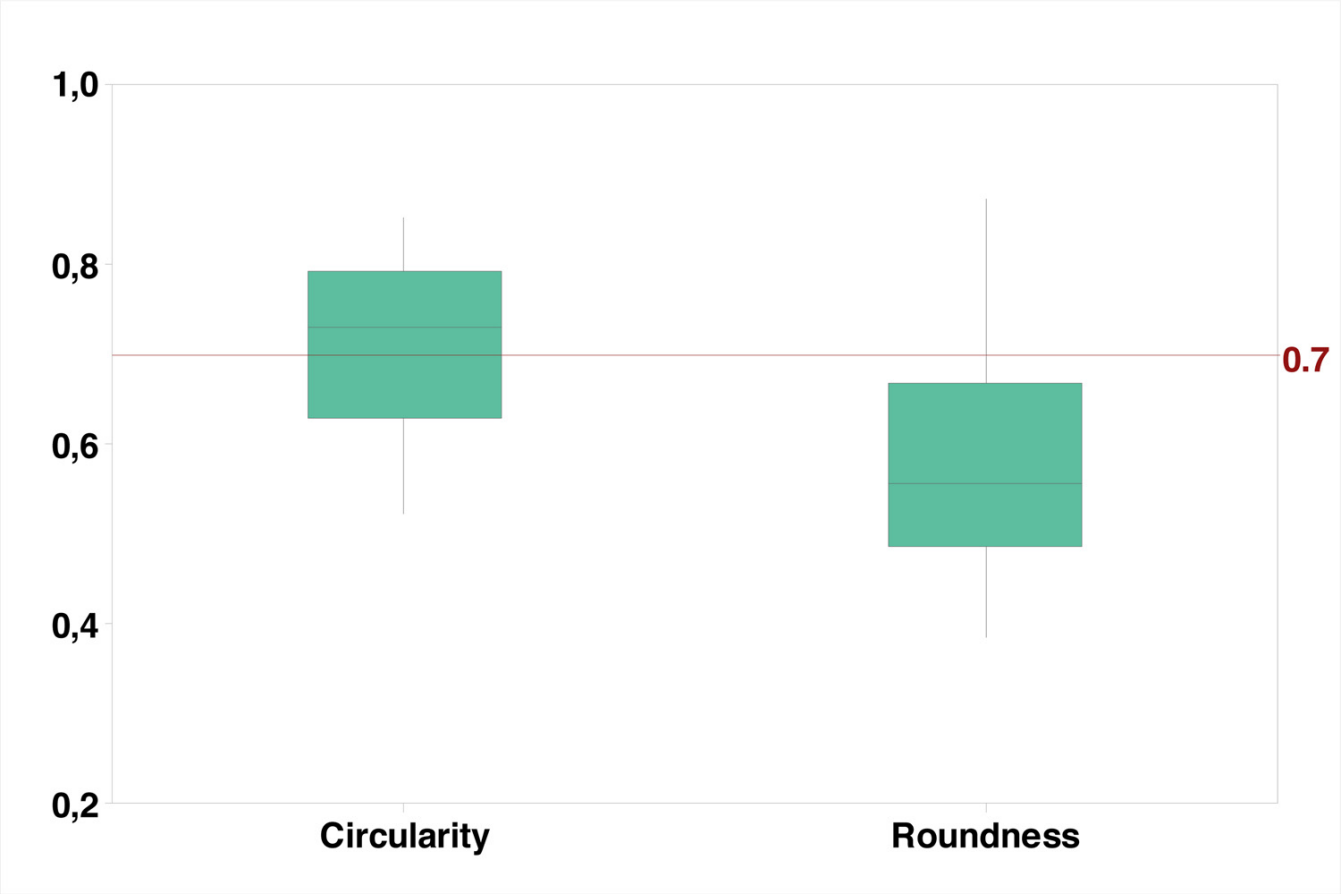
Circularity and Roundness of biomass.

Fig. 9 shows that the granules formed had excellent circularity characteristics (0.63 to 0.79). However, the roundness parameter was below the protocol requirement (0.48 to 0.67). This is attributed to the constraints found in the aeration since, as the heavier granules accumulated at the bottom of the reactor, obstruction of the aerator was generated, causing a decrease in shear forces, which in turn limited the development of the more regular, spherical, and compact aerobic granules as claimed by Hailei et al. [18].

Although the established characteristics were not reached, the values found for roundness and circularity are similar to those in reports by different authors: 0.64 to 0.67 and 0.64 to 0.66 [32], 0.61 ± 0.17 and 0.64 ± 0.20 [27], and 0.6 to 0.70 for the two characteristics, respectively [12].

Based on the Kolmogorov-Smirnov test, it was determined that the set of equivalent diameters of the granular aggregates did not present a normal distribution, so a nonparametric Wilcoxon test was adapted to the characteristics of the dataset [48], [52]. The result showed that for the mean of the granular formations obtained at the end of the AGP test, the equivalent diameter was above 0.20 mm.

## Conclusions

The PGA protocol is a novel and practical tool to assess the granulation potential of an inoculum through gradual stimulation. The PGA protocol also offers a flexible configuration based on a set of ranges that allow the variation of the most representative parameters of the aerobic granulation technology. Therefore, its application at different scales and areas is possible, achieving results in a short period (7 days) and with few economic, physical, and temporal resources. The AGP Protocol can be used to identify and adjust operating variables that lead to the optimization of existing wastewater treatment systems as well as the development of designs for new systems.
